# Association between intracranial arterial stenosis severity and recurrent stroke risk in elderly ischemic stroke patients

**DOI:** 10.3389/fneur.2026.1795462

**Published:** 2026-05-18

**Authors:** Zhen Tao, Hongwei Guo, Yingying Liu, Jun-Bin Yin, Hu Huai Qiang, Haowen Lu

**Affiliations:** 1 No. 971 Hospital of the People's Liberation Army Navy, Qingdao, China; 2 Department of Neurology, The 960(th) Hospital of Joint Logistics Support, PLA, Jinan, China

**Keywords:** elderly patients, intracranial arterial stenosis, ischemic stroke, secondary prevention, stroke recurrence

## Abstract

**Background and aim:**

Intracranial arterial stenosis (ICAS) is a major cause of ischemic stroke in older adults and is associated with substantial risk of recurrent cerebrovascular events. Whether increasing ICAS severity confers incremental recurrence risk in medically treated elderly patients in routine clinical practice remains incompletely characterized. This study aimed to investigate the association between intracranial arterial stenosis severity and 12-month recurrent stroke risk in elderly patients with ischemic stroke.

**Methods:**

This single-center retrospective cohort study screened 614 consecutive patients aged 65 years or older who were admitted with acute ischemic stroke between January 2022 and December 2023. After predefined exclusions, 527 patients were included in the final analysis. Intracranial arterial stenosis was assessed using computed tomography angiography or magnetic resonance angiography and categorized as none-to-mild (<50%; including no stenosis), moderate (50–69%), or severe (≥70%) according to the highest-grade lesion. The primary outcome was recurrent ischemic stroke within 12 months. Univariable and multivariable logistic regression models were used to evaluate independent predictors of recurrence.

**Results:**

Among the 527 included patients, recurrent ischemic stroke occurred in 86 (16.3%) during 12-month follow-up. Recurrence rates increased stepwise across stenosis categories, occurring in 18/214 (8.4%) patients with none-to-mild stenosis, 29/173 (16.8%) with moderate stenosis, and 39/140 (27.9%) with severe stenosis (*p* < 0.001). After adjustment for age, sex, vascular risk factors, stroke subtype, baseline NIHSS score, and medication use, severe ICAS remained independently associated with recurrent stroke (adjusted OR 3.12, 95% CI 1.85–5.26, *p* < 0.001); moderate stenosis was also independently associated with recurrence (adjusted OR 1.96, 95% CI 1.04–3.69, *p* = 0.037).

**Conclusion:**

In elderly patients with ischemic stroke, greater intracranial arterial stenosis severity is independently associated with higher 12-month recurrence risk despite contemporary medical management. These findings support early vascular imaging and risk stratification in elderly patients with ischemic stroke.

## Introduction

Ischemic stroke remains a leading cause of disability and mortality worldwide, particularly among elderly populations, in whom both incident and recurrent events contribute substantially to functional decline and healthcare burden (1, 2). Recurrent stroke is associated with worse functional outcomes, higher mortality, and greater long-term resource utilization than first-ever events, making risk stratification after the index event a central component of secondary prevention (3, 4).

Intracranial arterial stenosis (ICAS) is one of the most common and clinically important mechanisms underlying ischemic stroke, especially in Asian populations and in older adults with atherosclerotic vascular disease (5, 6). Prior studies have shown that ICAS is associated with impaired distal perfusion, artery-to-artery embolism, and recurrent ischemic events (5–7). While ICAS is widely recognized as a recurrence risk factor, the prognostic implications of different degrees of stenosis severity remain incompletely characterized in real-world cohorts of elderly patients. Importantly, prior cohort studies and randomized-trial datasets have shown that recurrent stroke risk remains clinically important in patients with symptomatic moderate-to-severe ICAS, particularly when stenosis exceeds 70%, despite aggressive medical management (6, 8–10).

Traditionally, clinical assessment of ICAS has focused on its presence or absence, with less attention to the incremental prognostic value of stenosis severity. Advances in noninvasive vascular imaging, including computed tomography angiography (CTA) and magnetic resonance angiography (MRA), have improved the reproducibility of grading intracranial arterial narrowing (11, 12). However, whether stratifying ICAS by severity adds clinically useful prognostic information beyond conventional vascular risk factors in elderly patients with ischemic stroke has not been fully clarified in routine clinical practice.

Against this background, the present study aimed to investigate the association between intracranial arterial stenosis severity and recurrent stroke risk in elderly patients with ischemic stroke in a real-world clinical setting. We focused on a single-center retrospective cohort of patients aged 65 years or older and examined 12-month recurrence according to none-to-mild, moderate, and severe ICAS categories while accounting for vascular risk factors, stroke subtype, and commonly used medical therapies.

## Materials and methods

### Study design and study population

**Figure 1:**
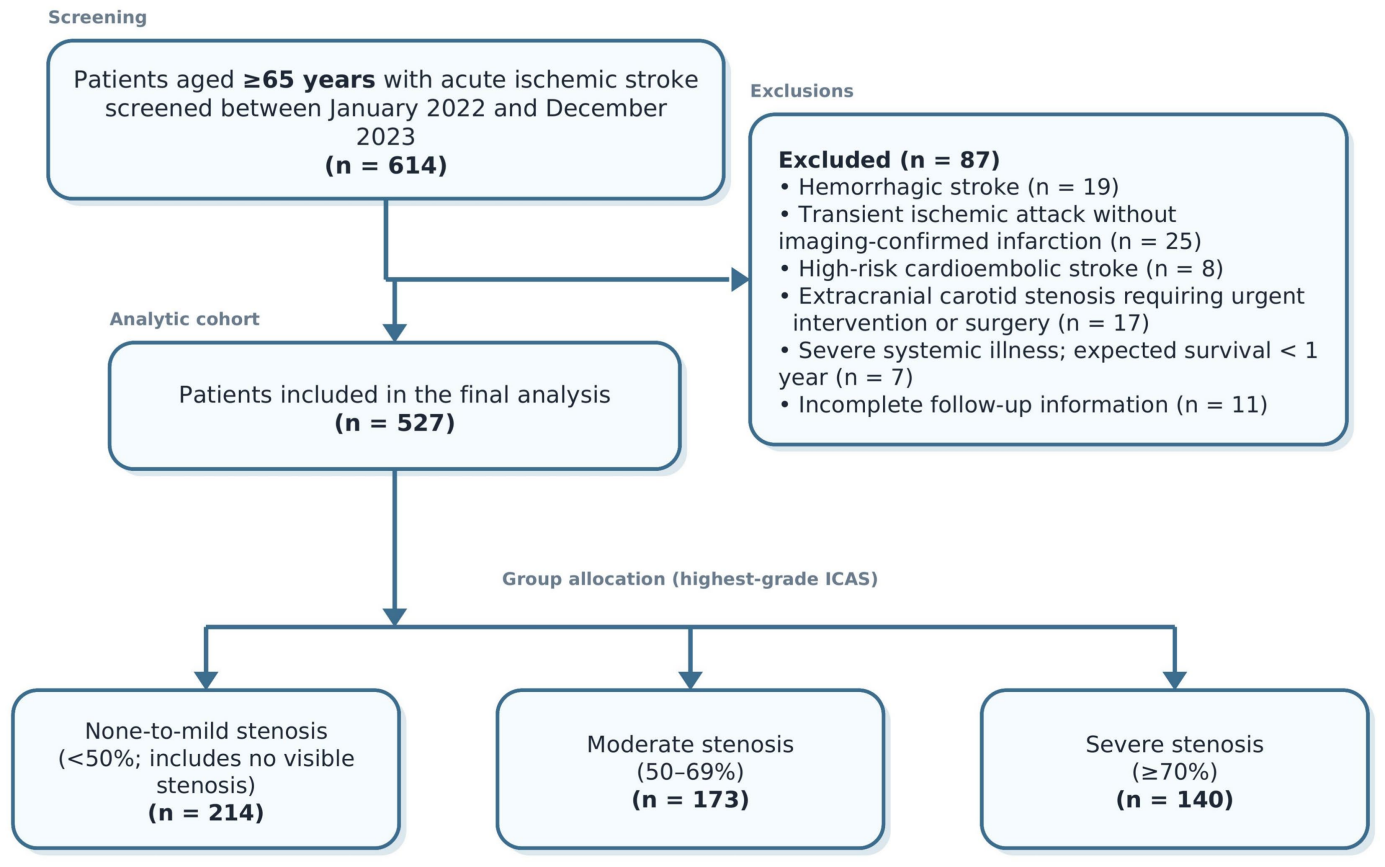
Flow chart of patient screening, exclusion, and group allocation. Patients without visible intracranial stenosis were included in the none-to-mild stenosis group (<50%).

This single-center retrospective cohort study was conducted at a tertiary teaching hospital. As summarized in Figure 1, 614 consecutive patients aged 65 years or older who were admitted with acute ischemic stroke between January 2022 and December 2023 were screened for eligibility. After exclusion of 19 patients with hemorrhagic stroke, 25 with transient ischemic attack without imaging-confirmed infarction, 8 with cardioembolic stroke due to high-risk cardiac sources, 17 with concomitant extracranial carotid stenosis requiring immediate intervention or surgery, 7 with severe systemic illness limiting life expectancy to less than 1 year, and 11 with incomplete follow-up information, 527 patients were included in the final analysis.

Ischemic stroke was diagnosed on the basis of acute focal neurological deficits consistent with cerebral infarction and confirmed by brain MRI or CT. Eligible patients were required to have intracranial vascular imaging during the index hospitalization and complete clinical, imaging, treatment, and follow-up data. Patients with hemorrhagic stroke, transient ischemic attack without infarction, high-risk cardioembolic stroke, concomitant extracranial carotid disease requiring urgent intervention, severe systemic illness with expected survival <1 year, or incomplete follow-up were excluded.

This study was approved by the institutional ethics committee and was conducted in accordance with the Declaration of Helsinki. The requirement for written informed consent was waived because of the retrospective study design and the use of anonymized routinely collected clinical data.

### Assessment of intracranial arterial stenosis

Intracranial arterial stenosis was evaluated using CTA or MRA performed during the index hospitalization. The prespecified arterial segments assessed were the intracranial internal carotid artery, middle cerebral artery M1 segment, anterior cerebral artery A1 segment, posterior cerebral artery P1 segment, intracranial vertebral artery V4 segment, and the basilar artery. Distal arterial branches were not systematically analyzed because of lower measurement reproducibility on routine retrospective CTA/MRA.

The degree of stenosis was determined according to the Warfarin–Aspirin Symptomatic Intracranial Disease (WASID) criteria and expressed as the percentage reduction in luminal diameter compared with the nearest normal segment (10). Patients were categorized according to the highest-grade lesion identified as having none-to-mild stenosis (<50%; including patients with no visible stenosis), moderate stenosis (50–69%), or severe stenosis (≥70%). Patients with more than one intracranial stenotic lesion were included, and the most severe lesion was used for group allocation. Imaging was independently reviewed by two experienced neurologists who were blinded to clinical outcomes, with disagreements resolved by consensus.

### Medical treatment and in-hospital management

All patients received guideline-based medical management during hospitalization. Antiplatelet therapy consisted of aspirin, clopidogrel, or dual antiplatelet therapy according to stroke mechanism, infarct pattern, bleeding risk, and treating physician judgment. Because this was a real-world elderly cohort rather than a trial population restricted to symptomatic severe ICAS, dual antiplatelet therapy was not uniformly prescribed to all patients with higher-grade stenosis.

Standard secondary prevention measures, including statin therapy, blood pressure control, and glycemic management, were implemented according to contemporary practice. Intravenous tirofiban was administered in selected patients with high thrombotic burden or progressive neurological deterioration. Edaravone dexborneol and butylphthalide were used as adjunctive acute-phase therapies according to local clinical practice and physician judgment. No patients underwent intracranial angioplasty or stenting during the study period.

### Follow-up and outcome measures

Patients were followed for 12 months after discharge through routinely scheduled outpatient assessments and structured telephone interviews during the follow-up period, rather than by a single end-of-study contact only. When recurrent cerebrovascular events were suspected, hospital readmission records, emergency records, and repeat neuroimaging reports were reviewed whenever available.

The primary outcome was recurrent ischemic stroke within 12 months, defined as a new focal neurological deficit of presumed vascular origin lasting more than 24 h, or a shorter-lasting deficit accompanied by new infarction on neuroimaging, occurring after the index event. Secondary outcomes were time to recurrence, unfavorable functional outcome at 12 months (modified Rankin Scale score >2), all-cause mortality during follow-up, hemorrhagic complications, and recurrent hospitalization for cerebrovascular events.

Recurrent events were adjudicated independently by two neurologists who were blinded to intracranial stenosis severity. Adjudication was based on follow-up interview data, available outpatient and inpatient medical records, emergency visit documentation, and repeat CT/MRI findings. Disagreements were resolved by consensus.

### Data collection

Clinical data were extracted from the hospital electronic medical record system and included demographic characteristics, vascular risk factors, stroke subtype, laboratory parameters, imaging findings, and medication use. Variables collected included age, sex, hypertension, diabetes mellitus, hyperlipidemia, smoking history, prior stroke, atrial fibrillation, coronary artery disease, baseline National Institutes of Health Stroke Scale (NIHSS) score, intracranial stenosis severity, and in-hospital treatments.

### Statistical analysis

Continuous variables were presented as mean ± standard deviation or median with interquartile range, and comparisons among groups were performed using one-way analysis of variance or the Kruskal–Wallis test as appropriate. Categorical variables were expressed as counts and percentages and compared using the chi-square test or Fisher’s exact test.

The primary endpoint was 12-month recurrent ischemic stroke status. Because exact censoring times for all nonrecurrent patients were not uniformly available in the retrospectively assembled dataset, time-to-event survival analyses were not retained. Accordingly, univariable and multivariable logistic regression models were used to estimate odds ratios (ORs) and 95% confidence intervals (CIs) for recurrent stroke. Variables with *p* < 0.10 in univariable analysis or deemed clinically relevant *a priori* were entered into the multivariable model.

Secondary binary endpoints were analyzed using chi-square or Fisher’s exact tests. Time to recurrence was summarized descriptively among patients with recurrent stroke and compared across groups using one-way analysis of variance. A two-sided *p* value <0.05 was considered statistically significant. All statistical analyses were performed using SPSS version 26.0 (IBM Corp., Armonk, NY, USA).

## Results

### Baseline characteristics of the study population

Of the 614 elderly patients screened during the study period, 527 met the study criteria and were included in the final analysis (Figure 1). The mean age of the cohort was 72.6 ± 5.8 years, and 312 patients (59.2%) were male. According to intracranial arterial stenosis severity, patients were classified into the none-to-mild stenosis group (<50%, *n* = 214), the moderate stenosis group (50–69%, *n* = 173), and the severe stenosis group (≥70%, *n* = 140).

Baseline characteristics are summarized in Table 1. Several demographic and vascular risk factor distributions were similar across groups, including sex, hypertension, hyperlipidemia, smoking history, atrial fibrillation, coronary artery disease, and aspirin or clopidogrel use. However, diabetes mellitus, prior ischemic stroke, baseline NIHSS score, large-artery atherosclerosis subtype, white blood cell count, fasting glucose, dual antiplatelet therapy, and tirofiban use differed significantly across stenosis categories.

**Table 1:** Baseline characteristics of elderly ischemic stroke patients according to intracranial arterial stenosis severity (*n* = 527).

Variable	None-to-mild stenosis (<50%) (*n* = 214)	Moderate stenosis (50–69%) (*n* = 173)	Severe stenosis (≥70%) (*n* = 140)	*p* value
Age, years (mean ± SD)	72.1 ± 5.6	72.8 ± 5.9	73.2 ± 6.1	0.218
Male sex, *n* (%)	123 (57.5)	103 (59.5)	86 (61.4)	0.712
Hypertension, *n* (%)	161 (75.2)	136 (78.6)	115 (82.1)	0.193
Diabetes mellitus, *n* (%)	72 (33.6)	71 (41.0)	63 (45.0)	0.047
Hyperlipidemia, *n* (%)	98 (45.8)	86 (49.7)	74 (52.9)	0.326
Current or former smoking, *n* (%)	79 (36.9)	71 (41.0)	63 (45.0)	0.298
Prior ischemic stroke, *n* (%)	39 (18.2)	41 (23.7)	38 (27.1)	0.041
Atrial fibrillation, *n* (%)	28 (13.1)	26 (15.0)	24 (17.1)	0.512
Coronary artery disease, *n* (%)	44 (20.6)	41 (23.7)	37 (26.4)	0.364
Baseline NIHSS score (median, IQR)	4 (2–6)	5 (3–7)	6 (4–9)	<0.001
Large-artery atherosclerosis subtype, *n* (%)	82 (38.3)	93 (53.8)	96 (68.6)	<0.001
Small vessel occlusion subtype, *n* (%)	89 (41.6)	52 (30.1)	24 (17.1)	<0.001
Anterior circulation infarction, *n* (%)	134 (62.6)	114 (65.9)	96 (68.6)	0.496
White blood cell count, ×10^9^/L (mean ± SD)	7.1 ± 2.0	7.4 ± 2.2	7.8 ± 2.4	0.032
LDL cholesterol, mmol/L (mean ± SD)	2.61 ± 0.72	2.69 ± 0.76	2.78 ± 0.81	0.084
Fasting glucose, mmol/L (mean ± SD)	6.4 ± 1.9	6.8 ± 2.1	7.2 ± 2.4	0.015
Aspirin use, *n* (%)	162 (75.7)	133 (76.9)	108 (77.1)	0.964
Clopidogrel use, *n* (%)	96 (44.9)	81 (46.8)	68 (48.6)	0.781
Dual antiplatelet therapy, *n* (%)	58 (27.1)	57 (32.9)	52 (37.1)	0.046
Edaravone dexborneol use, *n* (%)	141 (65.9)	118 (68.2)	99 (70.7)	0.582
Butylphthalide use, *n* (%)	126 (58.9)	111 (64.2)	95 (67.9)	0.143
Tirofiban use, *n* (%)	21 (9.8)	34 (19.7)	42 (30.0)	<0.001

SD, standard deviation; IQR, interquartile range; NIHSS, National Institutes of Health Stroke Scale; LDL, low-density lipoprotein.

### Stroke recurrence during follow-up

During the 12-month follow-up period, recurrent ischemic stroke occurred in 86 patients (16.3%). The recurrence rate increased progressively with the severity of intracranial arterial stenosis. Recurrent stroke was observed in 18 patients (8.4%) in the none-to-mild stenosis group, 29 patients (16.8%) in the moderate stenosis group, and 39 patients (27.9%) in the severe stenosis group (*p* < 0.001).

Patients with more severe intracranial stenosis also experienced earlier recurrence among those with recurrent stroke. The mean time to recurrence was 7.8 ± 2.6 months in the none-to-mild group, 6.3 ± 2.9 months in the moderate group, and 5.1 ± 3.1 months in the severe group (*p* < 0.001; Table 2).

**Table 2:** Clinical outcomes according to intracranial arterial stenosis severity.

Outcome	None-to-mild stenosis (<50%) (*n* = 214)	Moderate stenosis (50–69%) (*n* = 173)	Severe stenosis (≥70%) (*n* = 140)	*p* value
Recurrent ischemic stroke, *n* (%)	18 (8.4)	29 (16.8)	39 (27.9)	<0.001
Time to recurrence, months (mean ± SD)	7.8 ± 2.6	6.3 ± 2.9	5.1 ± 3.1	<0.001
Unfavorable functional outcome (mRS > 2), *n* (%)	61 (28.5)	67 (38.7)	69 (49.3)	<0.001
All-cause mortality during follow-up, *n* (%)	9 (4.2)	11 (6.4)	14 (10.0)	0.061
Hemorrhagic complications, *n* (%)	7 (3.3)	8 (4.6)	9 (6.4)	0.392
Recurrent hospitalization for cerebrovascular events, *n* (%)	22 (10.3)	31 (17.9)	37 (26.4)	<0.001

SD, standard deviation; mRS, modified Rankin Scale. Time to recurrence was calculated among patients with recurrent ischemic stroke.

### Association between intracranial stenosis severity and recurrent stroke

Univariable logistic regression identified older age, diabetes mellitus, prior ischemic stroke, higher baseline NIHSS score, large-artery atherosclerosis subtype, and moderate or severe ICAS as factors associated with recurrent ischemic stroke (Table 3).

**Table 3:** Univariable logistic regression analysis for recurrent ischemic stroke.

Variable	OR	95% CI	*p* value
Age (per year increase)	1.05	1.01–1.09	0.014
Male sex	1.12	0.71–1.78	0.628
Hypertension	1.29	0.76–2.18	0.342
Diabetes mellitus	1.83	1.14–2.95	0.012
Hyperlipidemia	1.21	0.76–1.94	0.414
Smoking history	1.46	0.92–2.31	0.109
Prior ischemic stroke	1.91	1.12–3.24	0.017
Atrial fibrillation	1.38	0.75–2.55	0.301
Baseline NIHSS score (per point)	1.18	1.11–1.25	<0.001
Large-artery atherosclerosis subtype	2.44	1.50–3.96	<0.001
Moderate ICAS (50–69%)	2.20	1.18–4.11	0.013
Severe ICAS (≥70%)	4.27	2.36–7.74	<0.001
Dual antiplatelet therapy	0.78	0.48–1.26	0.308
Edaravone dexborneol use	0.91	0.57–1.46	0.703
Butylphthalide use	0.87	0.54–1.39	0.560
Tirofiban use	1.39	0.84–2.30	0.198

OR, odds ratio; CI, confidence interval; NIHSS, National Institutes of Health Stroke Scale; ICAS, intracranial arterial stenosis.

In the multivariable logistic regression model adjusting for age, diabetes mellitus, prior ischemic stroke, baseline NIHSS score, large-artery atherosclerosis subtype, stenosis severity, and medication variables, severe intracranial arterial stenosis remained independently associated with recurrent stroke (adjusted OR 3.12, 95% CI 1.85–5.26, *p* < 0.001). Moderate stenosis was also independently associated with recurrence compared with the none-to-mild stenosis group (adjusted OR 1.96, 95% CI 1.04–3.69, *p* = 0.037; Table 4). Dual antiplatelet therapy, edaravone dexborneol, and butylphthalide were not significantly associated with 12-month recurrent stroke in the adjusted model.

**Table 4:** Multivariable logistic regression analysis for recurrent ischemic stroke.

Variable	Adjusted OR	95% CI	*p* value
Age (per year increase)	1.04	1.00–1.08	0.038
Diabetes mellitus	1.62	1.01–2.59	0.044
Prior ischemic stroke	1.71	1.01–2.89	0.046
Baseline NIHSS score	1.15	1.08–1.22	<0.001
Large-artery atherosclerosis subtype	1.89	1.10–3.24	0.021
Moderate ICAS (50–69%)	1.96	1.04–3.69	0.037
Severe ICAS (≥70%)	3.12	1.85–5.26	<0.001
Dual antiplatelet therapy	0.81	0.49–1.35	0.423
Edaravone dexborneol use	0.94	0.58–1.53	0.803
Butylphthalide use	0.88	0.54–1.45	0.620

OR, odds ratio; CI, confidence interval; NIHSS, National Institutes of Health Stroke Scale; ICAS, intracranial arterial stenosis.

### Secondary clinical outcomes

In unadjusted comparisons, secondary outcomes are summarized in Table 2. Patients with severe intracranial arterial stenosis had a higher proportion of unfavorable functional outcomes at 12 months, defined as modified Rankin Scale score >2, than those in the none-to-mild stenosis group. All-cause mortality during follow-up was numerically higher in the severe stenosis group, although this difference did not reach statistical significance.

No significant differences were observed among the groups in terms of hemorrhagic complications. Recurrent hospitalization for cerebrovascular events increased across the three stenosis categories (Table 2).

## Discussion

In this retrospective cohort of 527 elderly patients with ischemic stroke, we found a graded association between intracranial arterial stenosis severity and 12-month recurrent ischemic stroke. Recurrence increased stepwise from 8.4% in patients with none-to-mild stenosis to 16.8% in those with moderate stenosis and 27.9% in those with severe stenosis. After multivariable adjustment, both moderate and severe ICAS remained independently associated with recurrent stroke, with the strongest association observed for severe stenosis.

These findings are concordant with prior evidence showing that greater intracranial atherosclerotic burden and higher-grade stenosis are associated with increased recurrent risk (6, 7, 9, 10). In particular, the CICAS study and subsequent evidence synthesis in the AAN practice advisory both identified higher recurrence risk in patients with stenosis >70% compared with those with 50–69% stenosis (6, 9). Our study adds to this literature by focusing specifically on patients aged 65 years or older in a real-world hospital cohort, a population that is often underrepresented in trial-based analyses and may have a different balance of comorbidity, frailty, and treatment tolerance.

The biological plausibility of this association is strong. More severe ICAS may reflect greater plaque burden, unstable atherosclerotic morphology, impaired downstream perfusion, and a higher likelihood of artery-to-artery embolism or hemodynamic compromise (5, 13). The observation that moderate stenosis also carried higher recurrence risk than none-to-mild stenosis suggests that the 50–69% range should not automatically be considered low risk in elderly patients.

At the same time, our results should not be interpreted as evidence for or against the efficacy of any specific medical therapy, because treatment allocation was nonrandomized and confounded by clinical indication. Similarly, our data do not support an inference that endovascular intervention should be pursued on the basis of stenosis severity alone. Randomized trials such as SAMMPRIS did not demonstrate benefit for routine stenting over aggressive medical therapy in symptomatic ICAS (8). Rather, the present findings identify a subgroup of elderly patients who may warrant closer surveillance, tighter vascular risk-factor control, and careful follow-up under contemporary secondary prevention.

The relatively modest use of dual antiplatelet therapy, even in higher-grade stenosis, likely reflects real-world prescribing in elderly patients, including concern about bleeding risk, infarct size, comorbidity burden, and the fact that this cohort was not limited to trial-eligible symptomatic severe ICAS (14, 15). This is an important contextual feature of the study and may partly explain differences between routine practice and protocol-driven trial populations.

With respect to secondary outcomes, patients with severe ICAS had worse unadjusted functional outcomes at follow-up. This finding should be interpreted cautiously, however, because the functional outcome analysis was not adjusted for baseline differences and patients with severe stenosis also had higher baseline NIHSS scores, more prior stroke, and a higher proportion of large-artery atherosclerotic stroke subtype.

Our study has several limitations. First, this was a retrospective single-center study, so residual confounding and limited generalizability are unavoidable. Second, group allocation was based on the highest-grade lesion in patients with multiple intracranial stenoses; lesion multiplicity and total atherosclerotic burden were not modeled separately. Third, CTA/MRA-based grading may be less precise than digital subtraction angiography. Fourth, treatment duration, adherence, and changes in secondary prevention after discharge were not fully captured. Finally, edaravone dexborneol and butylphthalide are not standard stroke therapies worldwide, which may limit the external generalizability of our findings.

## Conclusion

In conclusion, greater intracranial arterial stenosis severity was independently associated with higher 12-month recurrent stroke risk in elderly patients with ischemic stroke in this real-world cohort. Assessment of stenosis severity may help identify older patients at increased risk of recurrence and support individualized follow-up and secondary prevention strategies.

## Data Availability

The original contributions presented in the study are included in the article/supplementary material, further inquiries can be directed to the corresponding author.
